# Clinically relevant radioresistant rhabdomyosarcoma cell lines: functional, molecular and immune-related characterization

**DOI:** 10.1186/s12929-020-00683-6

**Published:** 2020-08-27

**Authors:** Francesco Petragnano, Ilaria Pietrantoni, Simona Camero, Silvia Codenotti, Luisa Milazzo, Francesca Vulcano, Giampiero Macioce, Ilenia Giordani, Paolo Tini, Sara Cheleschi, Giovanni Luca Gravina, Claudio Festuccia, Alessandra Rossetti, Simona Delle Monache, Alessandra Ordinelli, Carmela Ciccarelli, Annunziata Mauro, Barboni Barbara, Cristina Antinozzi, Amalia Schiavetti, Roberto Maggio, Luigi Di Luigi, Antonella Polimeni, Cinzia Marchese, Vincenzo Tombolini, Alessandro Fanzani, Nicola Bernabò, Francesca Megiorni, Francesco Marampon

**Affiliations:** 1 Department of Biotechnological and Applied Clinical Sciences, University of L’Aquila, L’Aquila, Italy; 2 Department of Maternal, Infantile, and Urological Sciences, “Sapienza” University of Rome, Rome, Italy; 3 Department of Molecular and Translational Medicine, Division of Biotechnology, University of Brescia, Brescia, Italy; 4 Department of Oncology and Molecular Medicine, Istituto Superiore di Sanità, Viale Regina Elena, Rome, Italy; 5 Department of Anatomy, Histology, Forensic Medicine and Orthopedics, Section of Histology and Medical Embryology, “Sapienza” University, Rome, Italy; 6 Sbarro Health Research Organization, Temple University, Philadelphia, PA USA; 7 Unit of Radiation Oncology, University Hospital of Siena, Siena, Italy; 8 Department of Medicine, Surgery and Neuroscience, Rheumatology Unit, University of Siena, Policlinico Le Scotte, Siena, Italy; 9 Faculty of Bioscience and Technology for Food, Agriculture and Environment, University of Teramo, Teramo, Italy; 10 Unit of Endocrinology, Department of Movement, Human and Health Sciences, University of Rome “Foro Italico”, Rome, Italy; 11 Department of Oral and Maxillo-Facial Sciences, Sapienza University of Rome, Rome, Italy; 12 Department of Experimental Medicine, Sapienza University of Rome, Rome, Italy; 13 Department of Radiotherapy, Policlinico Umberto I, “Sapienza” University of Rome, Rome, Italy

**Keywords:** Rhabdomyosarcoma, Radiotherapy, Radioresistance, Radiobiology, Immunoescape

## Abstract

**Background:**

The probability of local tumor control after radiotherapy (RT) remains still miserably poor in pediatric rhabdomyosarcoma (RMS). Thus, understanding the molecular mechanisms responsible of tumor relapse is essential to identify personalized RT-based strategies. Contrary to what has been done so far, a correct characterization of cellular radioresistance should be performed comparing radioresistant and radiosensitive cells with the same isogenic background.

**Methods:**

Clinically relevant radioresistant (RR) embryonal (RD) and alveolar (RH30) RMS cell lines have been developed by irradiating them with clinical-like hypo-fractionated schedule. RMS-RR cells were compared to parental isogenic counterpart (RMS-PR) and studied following the radiobiological concept of the “6Rs”, which stand for repair, redistribution, repopulation, reoxygenation, intrinsic radioresistance and radio-immuno-biology.

**Results:**

RMS-RR cell lines, characterized by a more aggressive and in vitro pro-metastatic phenotype, showed a higher ability to i) detoxify from reactive oxygen species; ii) repair DNA damage by differently activating non-homologous end joining and homologous recombination pathways; iii) counteract RT-induced G2/M cell cycle arrest by re-starting growth and repopulating after irradiation; iv) express cancer stem-like profile. Bioinformatic analyses, performed to assess the role of 41 cytokines after RT exposure and their network interactions, suggested TGF-β, MIF, CCL2, CXCL5, CXCL8 and CXCL12 as master regulators of cancer immune escape in RMS tumors.

**Conclusions:**

These results suggest that RMS could sustain intrinsic and acquire radioresistance by different mechanisms and indicate potential targets for future combined radiosensitizing strategies.

## Background

Rhabdomyosarcoma (RMS) is the most common soft-tissue sarcoma in childhood. Two main histotypes characterize RMS: alveolar (ARMS), the highest-grade tumor, and embryonal (ERMS), the most frequent type. They respectively express more frequently the pro-oncogenic fusion proteins encoding paired box protein 3/encoding forkhead box protein O1 (PAX3/FOXO1) or multiple numerical chromosome aberrations and RAS (Rat Sarcoma viral oncogene homolog) mutations. However, independently from the genetic background, the molecular mechanisms responsible of RMS development, progression and resistance to therapies commonly converge on the aberrant activation of specific pathways, including those involved in the repair of damaged DNA [1]. The current standard of care for early and locally advanced RMS includes surgical resection combined to chemotherapy (CHT) and/or radiotherapy (RT) [1, 2]. RT is crucial for local control at primary and metastatic sites in pediatric RMS, preventing in-field progression in both cases. However, treatment frequently fails resulting in disease progression [1, 2].

RT, by using ionizing radiations (IR), is able to kill cancer cells directly by inducing DNA double strand breaks (DSBs) [3], and indirectly by promoting immunogenic cell death (ICD), which consists of recruiting the host immune system [4] preferentially by the release of several mediators, including cytokines [5]. However, cancer cells can efficiently escape from RT-induced cell death trough different mechanisms, such as resistance to apoptosis, high DNA repair capacity, antioxidant capacities and ICD escape [6]. Notably, radioresistance has been shown to be higher in cancer stem cells (CSCs) [7], known to be the critical driving force of cancer and the real target of any antitumoral therapeutic approach [8].

Notwithstanding several studies have identified molecular mechanisms implicated in radioresistance, the largest part has been performed by using cancer cells with different grade of radio-resistance, genetic backgrounds and origins. On the other hand, as recently suggested, biological systems able to compare radioresistant and sensitive cells with the same isogenic background should be preferred [6, 9].

In this study, we present novel clinically radioresistant RMS (RMS-RR) cancer cell lines, obtained by irradiating ERMS RD and ARMS RH30 cells [10] with a hypo-fractionated-based schedule of RT similar to that used in the clinical practice. The radiobiology characterization of these cell lines, comparing them to their isogenic background, has provided a variety of valuable information that might be translated into meaningful clinical applications in order to improve the therapeutic efficiency of RT, alone and in combination with targeted therapies or immunotherapy, against RMS tumors.

## Methods

### Cell culture and in vitro assays

Human RMS cell lines, RD (ERMS) and RH30 (ARMS) [10], both from American Type Culture Collection,

were respectively cultured in Dulbecco’s modified Eagle’s and RPMI medium containing 10% fetal calf serum (Hyclone, Logan UT) and supplemented with glutamine and gentamycin (GIBCO-BRL Gaithersburg, MD). Human umbilical vein endothelial cells, HUVECs (Clonetics, San Diego, California, USA) were cultured in endothelial cell basal medium (EBM-2; Clonetics) supplemented with 2% of fetal calf serum (FCS; Clonetics) and endothelial growth medium (EGM2; Clonetics). Multipotent mesenchymal stromal cells (MSC) from Wharton’s jelly of umbilical cord [11], were cultured in Dulbecco’s modified Eagle’s medium containing 10% fetal calf serum (Hyclone) and supplemented with glutamine and gentamycin (GIBCO-BRL). Cells were incubated at 37 °C in 5% CO2. Medium was replaced every 3 days. Cells from passages 5–7 were used for all the experiments. DNA profiling, using the GenePrint 10 System (Promega Corporation, Madison, WI, USA), was carried out to authenticate cells by comparing the DNA profile of our cell cultures with those found in GenBank. MycoFluor™ Mycoplasma Detection Kit Invitrogen™ was used.

### Irradiation of cells

Radiation was delivered at room temperature using an x-6 MV photon linear accelerator. The total single dose was delivered with a dose rate of 2Gy/min using a source-to-surface distance (SSD) of 100 cm. Doses of 200 kV X-rays (Yxlon Y.TU 320; Yxlon, Copenhagen, Denmark) filtered with 0.5 mm Cu. The absorbed dose was measured using a Duplex dosimeter (PTW, Freiburg, Germany). To select clinically relevant radioresistant (RR) cell lines, 24 h after irradiation, 30% of irradiated cells were re-seeded and the next irradiation was repeated when a confluence of 80% was reached again. This was repeated for 6 times to a final equivalent dose (EQD_2_) of 66 Gy (α/β ratio for RMS = 2.8 Gy [12], BED = 113.14 Gy) used in the clinical practice [2].

### Clonogenic survival assay

For clonogenic survival, exponentially growing cells (70% confluence) were cultured in regular media and, 24 h after plating, irradiated at room temperature with increasing doses of radiation (0–6 Gy) by using an X-ray linear accelerator (dose rate of 200 cGy/min). Non-irradiated controls were handled identically to the irradiated cells, with the exception of the radiation exposure. After treatment, cells were diluted at the appropriate concentration (1000 cells), re-seeded into a new 100-mm tissue culture dish (in triplicate) and incubated for 14 days. At day 14, culture medium was removed and colonies were fixed with methanol:acetic acid (10:1, v/v) and stained with crystal violet. Colonies containing > 50 cells were counted. Plating efficiency (PE) was calculated as the number of colonies observed/the number of cells plated; the surviving fraction (SF) was calculated as follows: colonies counted/cells seeded x (PE/100). The mean inactivation dose was calculated according to the method already described [13], and the cell survival enhancement ratio (ER) was calculated as the ratio of the mean inactivation dose under controlled conditions, divided by the mean inactivation dose after drug exposure, as already described [14].

### Cell proliferation assay and FACS analysis

Cells from adherent and suspension cultures were counted using a hemocytometer and tested for exclusion of trypan blue dye. Data are expressed as average of triplicates + standard error. For FACS analysis, cells were harvested by trypsin-EDTA and washed; pellets were resuspended in 0.3 ml 50% FCS in PBS, additioned with 0.9 ml 70% ethanol, and left overnight in the dark at + 4 °C before flow cytometry (Coulter Epics XL Flow Cytometer, Beckman Coulter CA, USA). Propidium iodide (PI) staining was used for cell cycle analysis.

### Annexin V/PI staining assay

Cells were seeded in 6-well plate at a density of 2 × 10^5^/well and allowed to adhere overnight. Treatment and incubation were performed as required. Cell apoptosis was determined by Annexin V/PI labeling according to the manufacturer’s protocol (Invitrogen). The early and late apoptotic cells were detected using a flow cytometry instrument (BD FACS CantoTM, BD Biosciences, San Jose, CA, United States).

### Sphere and tube formation assay

Sphere-forming cells were obtained by culturing RMS cells in anchorage-independent conditions (low attachment flasks or plates, Nunc) in SC-medium, consisting in DMEM:F12 medium (Gibco-Invitrogen) with progesterone (2 μM), putresceine (10 μg/ml), sodium selenite (30 nM), apo-transferrin (100 μg/ml) and insulin (50 mg/ml) (all from Sigma-Aldrich). Fresh human epidermal growth factor (20 ng/ml) and fibroblast growth factor (20 ng/ml) (PeproTech, London, UK) were added twice/week until cells formed floating spheres. To evaluate the primary sphere formation, cells from sub-confluent (70–80%) monolayer cultures were plated at a density of 100, 500 or 1000 cells in a 24-well culture plate (Corning Inc., Corning, NY, USA). For sphere formation assay, the number of primary tumorspheres was counted. The tube formation assay was carried out by using the in vitro Matrigel assay kit (Chemicon, Millipore) following the manufacturer’s instructions by coating 15-well micro-slides (10 μl/well) of IBIDI (Munich, Germany).

### Mitochondrial superoxide anion (·O_2_−) production

RMS cell lines were seeded in 6-well plates at a starting number of 6 × 10^4^ cells/well for 24 h in regular medium and, then, irradiated. Immediately and 12 h after radiation exposure, flow cytometry analysis was performed. Medium was discarded and cells were incubated in Hank’s Balanced Salt Solution (HBSS) (Sigma-Aldrich, Milan, Italy) and MitoSOX Red (Thermo Fisher Scientific, Milan, Italy) for 15 min at 37 °C in dark to evaluate mitochondrial superoxide anion (·O2^−^) production. MitoSOX Red was dissolved in DMSO at the final concentration of 5 μM. Cells were then harvested by trypsin, collected into cytometry tubes and centrifuged at 1500 rpm for 10 min. Besides, 1 × 10^4^ cells per assay were resuspended in saline solution and analyzed by flow cytometry. Data were analyzed with CellQuest software (Becton Dickinson) and results were represented as median of fluorescence (AU).

### RNA isolation and quantitative real-time PCR

Total RNA was isolated by tumor cells by using 1 ml of TRIzol LS reagent (Invitrogen, Carlsbad, CA) per 50–100 mg of sample according to the manufacturer’s protocol. RNA concentration and purity were measured by NanoDrop 2000 (Thermo Fisher Scientific, Inc., Waltham, MA). Reverse transcription for target genes was performed by using QuantiTect Reverse Transcription Kit (Qiagen, Hilde, Germany), according to the manufacturer’s instructions. Target genes were analyzed by quantitative real-time PCR (qPCR), by using the following primers from Qiagen: SOD-2 (QT01008693), CAT (QT00079674), GPx4 (QT00067165), NRF2 (QT00027384) and β-Actin (ACTB) (QT00095431). Each sample was run in triplicate, in at least two independent experiments, on a StepOne Real Time System (Applied Biosystems) machine [15]. Relative quantification (RQ) of each mRNA in RR samples in comparison to PR-cells was calculated by the comparative Ct method (2^-ΔΔCt^), using the StepOne v2.3 software (Applied Biosystems). RQmax and RQmin, which are the maximum and minimum limits of the RQ values based on the standard error of the mean ΔCt values at 95% confidence interval, were used to plot error bars.

### Immunoblotting

Cells were lysed in 2% SDS containing 2 mM phenyl-methyl sulphonyl fluoride (PMSF) (Sigma), 10 μg/ml antipain, leupeptin and trypsin inhibitor, 10 mM sodium fluoride and 1 mM sodium orthovanadate (all from Sigma) and sonicated for 30 s. Proteins of whole cell lysates were assessed using the Lowry method [16], and equal amounts were separated on SDS-PAGE. The proteins were transferred to a nitrocellulose membrane (Schleicher & Schuell, BioScience GmbH, Germany) by electroblotting. The balance of total protein levels was confirmed by staining the membranes with Ponceau S (Sigma). Immunoblottings were performed with the following antibodies: Cdc25A (DCS-120), Cdk1 Antibody (AN21.2), Cyclin B1 (H^− 20^), p21^Waf1/Cip1^ (C^− 19^), p27^Kip1/Cip1^ (F^− 8^), c-Myc (9E10), N-Myc Antibody (NMYC-1), Ku70 Antibody (A-9), Ku80 Antibody (B-1), phospho-ATM (10H11.E12, Ser1981), ATM (H-248), DNA-PKCs (E-6), H2AX (C-20), phospho-VEGFR2 MoAb (pFlk-1), VEGFR2 (Flk-1), HIF-2α (EPAS-1), α-Tubulin (TU-02), goat anti-mouse IgG-HRP (sc-2005) and goat anti-rabbit IgG-HRP (sc-2004) by Santa Cruz Biotechnology; HIF-1α by Cell Signalling (Cell Signalling Technology, Inc.); Cyclin A1 (ab53699), phospho-DNA-PKCs (Thr2609) (10B1) by AbCam (Cambridge, UK). Protein signals were detected using Western Bright ECL kit (Advansta, Menlo Park, CA) and visualized by ChemiDoc XRS+ (Bio-Rad, Hercules, CA). Densitometry was performed to quantify changes in protein expression using the Image Lab5.1 software (Bio-Rad).

### Multiplex chemokine assay and TGF-β ELISA

Cytokines were assessed on cell culture supernatants by magnetic bead-based multiplex assay (Bio-Plex Pro™ Human Chemokine Panel, 40-Plex). Cytokines included were: CCL1, CCL2, CCL3, CCL7, CCL8, CCL11, CCL13, CCL15, CCL17,CCL19, CCL20, CCL21, CCL22, CCL23, CCL24, CCL25, CCL26,CCL27, CXCL1, CXCL2, CXCL5, CXCL6, CXCL8, CXCL9, CXCL10, CXCL11, CXCL12, CXCL13, CXCL16, CX3CL1, IL1, IL2, IL4, IL6, IL10, IL16, MIF, GMCSF, IFN- γ, TNF-α). Data acquisition was performed by Bio-Plex 200 System™ (Bio-Rad Laboratories, Inc.) which uses Luminex fluorescent-bead-based technology (Luminex). Data analysis was performed by Bio-Plex Manager™ 6.0 software (Bio-Rad Laboratories, Inc.). TGF-β was assessed by Human active TGF-β ELISA kit (R&D Systems, Minneapolis, MN, USA).

### Statistical analysis and data analysis

The results were expressed as the mean ± SD of three independent experiments, each performed in triplicate. Data normal distribution was confirmed by Shapiro–Wilk, D’Agostino and Pearson and Kolmogorov–Smirnov tests. Real-time PCR experiments were evaluated by one-way (ANOVA) with a Tukey’s post hoc test using 2^−ΔΔCt^ values for each sample. Flow cytometry data were analyzed by.

ANOVA with a Bonferroni post hoc test. All analyses were performed using the SAS System (SAS Institute Inc., Cary, NC, USA) and GraphPad Prism 6.1. A statistically significant effect was indicated by a *p* value < 0.05. Principal Component Analysis (PCA), performed with the Past3 software, has been applied to the study of chemokines expression. The Search Tool for the Retrieval of Interacting Genes/Proteins (STRING) [17] has been used to predict new molecular interactions possibly involved in cytokines network. Network visualizations have been realized and analyzed with Cytoscape 3.7.2, and the specific plugs-in Network Analyzer and Biological Network Gene Ontology (BINGO). Topological parameters assessed in this study are reported in Additional data 1.

## Results

### Development and onco-phenotypic characterization of clinically relevant radioresistant RMS cell lines

**Fig. 1:**
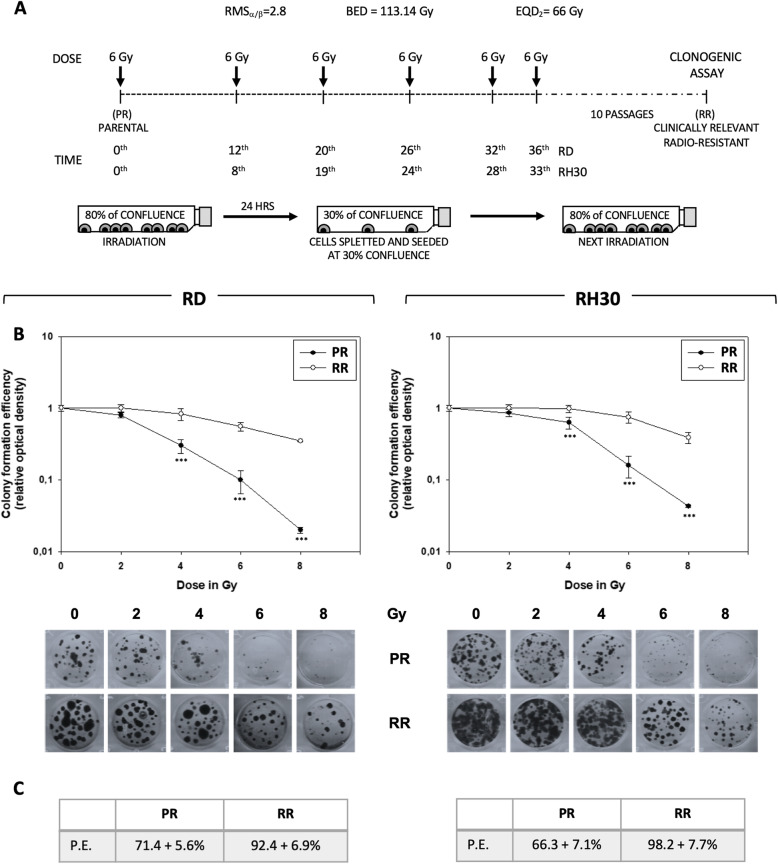
Development of clinically relevant radio-resistant cell line. **a** Representation of the radiation schedule used and the related radiobiological parameters. Growing RD and RH30 cells at 80% of confluence were irradiated with the dose of 6 Gy. 24 h after irradiation, 30% of irradiated cells were re-seeded and the next irradiation repeated when a confluence of 80% was reached again, this for 6 times to get a final equivalent dose (EQD2) to that reached with conventional fractionation of 66 Gy into daily dose of 2 Gy. **b** Upper Panel. Clonogenic assay of the parental (PR) and clinically relevant (RR) RD (Left Panel) and RH30 (Right Panel) lines with increasing dose of irradiation (2, 4, 6, 8 Gy). Data are expressed as relative optical density vs. non-irradiated cells, taken as 1. Representative crystal violet stained cultures 14 days after irradiation. **c** Percent of plating efficiency. Results represent the mean values of four independent experiments ± SD. Statistical significance: *p ≤ 0.05, **p ≤ 0.01, ***p ≤ 0.001 compared RMS-PR vs. RMS-RR

**Fig. 2:**
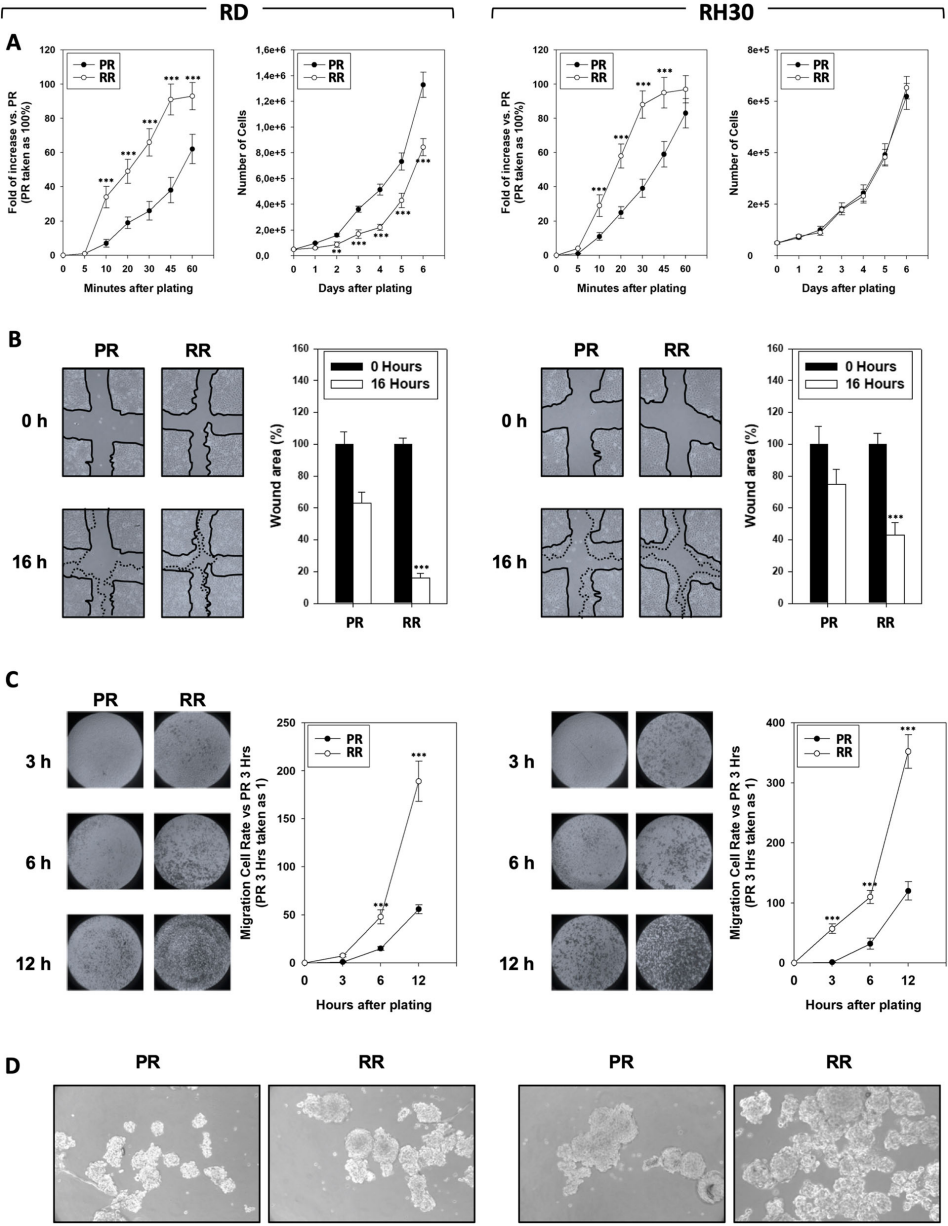
Onco-phenotype characterization of clinically relevant radio-resistant cell line. **a** Panels show the ability of RMS-PR and -RR to attach and spread (Left Panel) and proliferate (Right Panel) on a fibronectin coated plate. Data of attachment assay are expressed as fold of increase vs. non-irradiated cells, taken as 1. **b** Wound healing experiments in RMS-PR and RMS-RR cells. A scratch was made at time 0 and maintained or not for 16 h. The dotted lines represent the edges of the wound. Photographs (Left Panel) were taken under light microscope (10x magnification). The migration index was plotted in bar graphs as the % of wound area (Right Panel). **c** Matrigel invasion assay. Cells were allowed to invade for 24 h in serum-free medium. Pictures shown are the most representative from three independent experiments. The graph represents absorbance at 595 nm after incubation of the membranes with deoxycholic acid. Results represent the mean values of four independent experiments ± SD. Statistical significance: *p ≤ 0.05, **p ≤ 0.01, ***p ≤ 0.001 compared RMS-RR vs. RMS-PR. **d** Representative microphotographs of RMS-PR and RMS-RR cells after 14 days of incubation in stem cell medium

RT for RMS tumors usually provides 50/66 Gy in fractions of 2 Gy [2]. However, hypofractioned programs, single higher doses for a reduced number of fractions, are used to overcome the intrinsic radioresistance of RMS [18]. In order to generate clinically relevant radioresistant (RR) RMS cell lines, RD and RH30 cells were subjected to hypo-fractionated schedule based on the use of 6 fractions, each at 6 Gy. Since tumor cells in 2D are more sensitive to treatments than *in vivo* [19] and according to others already tested protocols [9], cells were re-irradiated when showed a recovery of proliferative potential, as summarized by the representation in Fig. 1a. Notably, time-intervals between subsequent irradiations progressively decreased, this suggesting the acquisition of a radioresistant phenotype by the cells (Fig. 1, Inter-fraction time). Clonogenic assays, performed by irradiating parental (PR) and RR RMS cells with increasing dose of RT (0–2–4-6-8 Gy), confirmed that colony formation ability resulted significantly increased in RR than PR cells. Moreover, when the maximum RT dose was used (8 Gy), few PR cells survived while a significant number of RR types was still present (Fig. 1b). RMS-RR cells also showed a higher plating efficiency, which was 92.4 ± 6.9% in RD-RR vs. 71.4 ± 5.6% in RD-PR and 98.2 ± 7.7% in RH30-RR vs. 66.3 ± 7.1% in RH30-PR (Fig. 1c). Onco-phenotypic characterization was then performed. The ability of RMS cells to adhere and grow up onto fibronectin-coated plates was assessed: RD- and RH30-RR, already after 10 min from plating, more efficiently adhered to substrate (Fig. 2a, left panel, RMS-RR vs. RMS-PR, 10 min), and differently from PR cells, reached a plateau after 60 min (Fig. 2a, left panel, RMS-RR vs. RMS-PR, 60 min). Once adhered, the proliferation rate was lower in RD-RR compared to RD-PR cells (Fig. 2a, right panel, RD-RR vs. RD-PR) while no substantial difference was described between RH30-PR and -RR cells (Fig. 2a, right panel, RH30-RR vs. RH30-PR). Scratch wound healing assays (Fig. 2b), in which the same fields of confluent cells were pictured immediately after the scratch (time 0 h) and again 16 h later, showed that RD-RR decreased the level of wound closure to 17.4 ± 4.1% vs. 64.3 ± 6.8% of RD-PR (Fig. 2b, RD, RR vs. PR), whilst RH30-RR to 41.2 ± 6.9% vs. 73.2 ± 8.6% of RH30-PR (Fig. 2b, RH30, RR vs. PR). Invasion capacity (Fig. 2c), measured 24 h after plating by assessing the ability of cancer cells to pass through a Matrigel-coated membrane, resulted increased by about 3.8 and 3.1-fold in RD-RR and RH30-RR cells, compared to the mocked RMS-PR controls (Fig. 2c, RMS, RR vs. PR). The ability to form floating rhabdo-spheres enriched in cancer stem-like cells (CSCs) [20] was also tested. When growth in non-adherent conditions and in the presence of stem cell (SC)-medium, RMS-RR cells formed rhabdo-spheres more efficiently than parental cells by 59.9 ± 12.4% in RD (Fig. 2d, RD, RR vs. PR) and 62.1 ± 8.3% in RH30 (Fig. 2d, RH30, RR vs. PR). No statistically significant differences were observed between RMS-PR and -RR cells about the ability to induce neo-angiogenesis and on the activation/expression status of pro-angiogenetic factors including VEGF receptor, HIF-1α and HIF-1β (Additional data 2).

### RMS-RR cells more efficiently than RMS-PR detoxify from ROS and repair DSBs

**Fig. 3:**
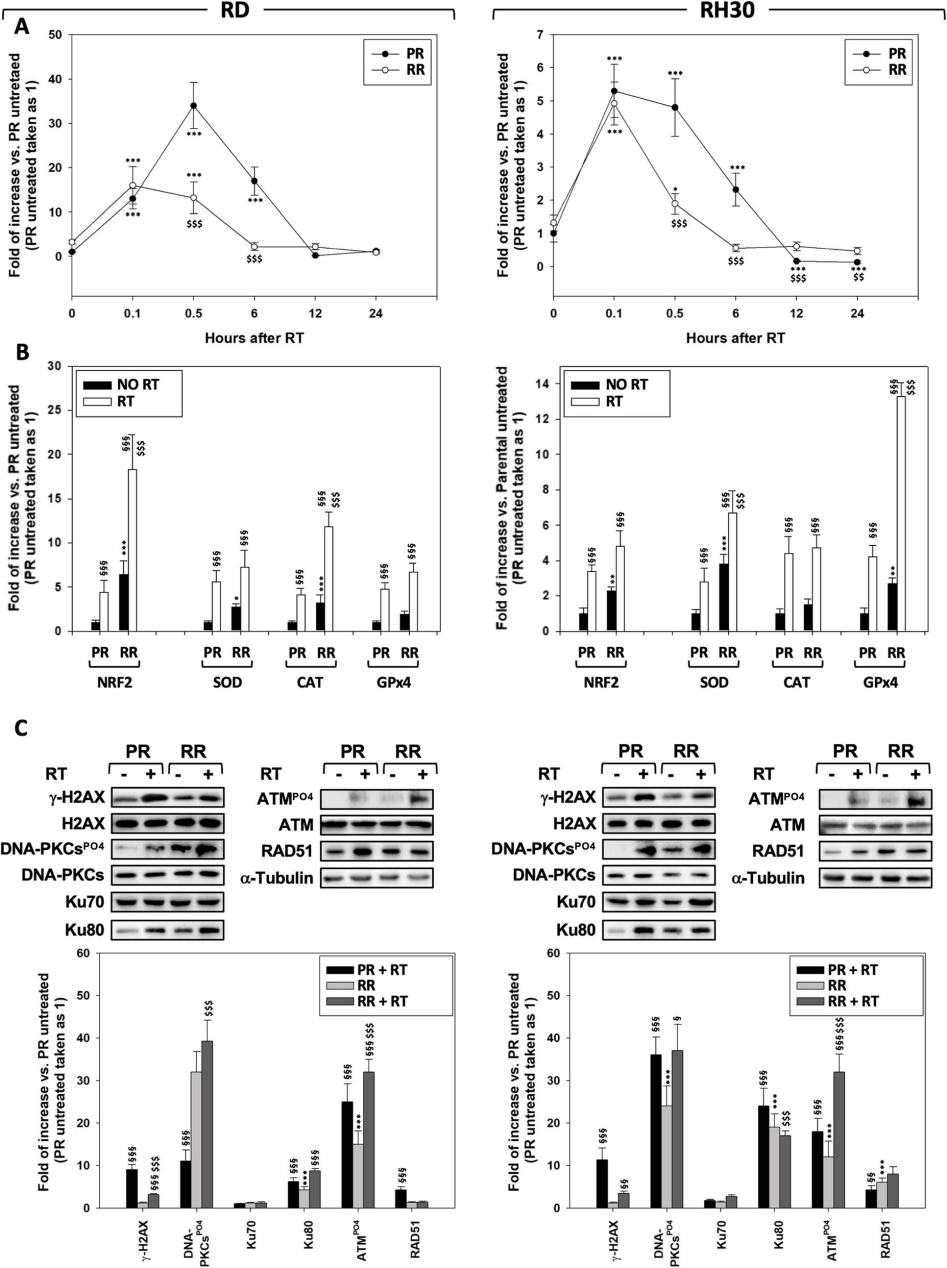
RMS-PR and -RR cells differently trigger anti-oxidant and DNA repair responsiveness after irradiation. **a** Mitochondrial superoxide anion production was assessed by MitoSox Red staining, 10 min (0.1), 30 min (0.5), 12 or 24 h after RT in RMS-PR and RMS-RR cells. **b** Gene expression of antioxidant enzymes nuclear factor erythroid 2-related factor (NRF2), superoxide dismutase (SOD-2), catalase (CAT) and glutathione peroxidase (GPx)-4 was investigated by real-time PCR, 12 h after RT. The gene expression was referenced to the ratio of the value of interest and basal conditions. The value of basal conditions was reported equal to 1. **c** Cell lysates from RMS-PR and RMS-RR cells untreated (−) or treated (+) with 6 Gy of irradiation collected 12 h after RT, were analyzed by immunoblotting with specific antibodies for indicated proteins; α-Tubulin expression shows the loading of samples. Western blot showed are representative of three different experiments. Statistical analyses: *p < 0.05, **p < 0.01, ***p < 0.001 RMS-RR NO RT vs. RMS-PR NO RT, ^§^p < 0.05, ^§§^p < 0.01, ^§§§^p < 0.001 RMS-PR RT vs. RMS-PR NO RT and RMS-RR RT vs. RMS-RR NO RT, ^$^p < 0.05, ^$$^p < 0.01, ^$$$^p < 0.001 RMS-RR RT vs. RMS-PR RT.

RT-mediated ROS production causes two-third of DSBs and cancer cells [3], including RMS [21, 22], frequently express aberrant levels of free radical scavenging systems that actively participated in promoting radioresistance mechanisms [23]. In order to characterize the antioxidant response of RMS-RR cell lines, mitochondrial ROS production was assessed 0.1, 0.5, 6 12 and 24 h after 6 Gy of RT, by measuring the superoxide anion production. As shown in Fig. 3a, IR rapidly induced ROS accumulation indistinctly in PR and RR RMS (Fig. 3a, 0.1 h, RD and RH30, RR + RT vs. PR + RT). However, half an hour after RT, RT-induced ROS accumulation still persisted in PR- but not in RR-RMS (Fig. 3a, 0.5 h, RD and RH30, RR + RT vs. PR + RT) that progressively recovered to basal levels earlier that PR (Fig. 3a, 6 h, RD and RH30, RR + RT vs. PR + RT). Data from q-PCR experiments showed that, compared to RMS-PR, RT more efficiently upregulated gene expression of NRF2 and CAT in RD-RR (Fig. 3b, RD, RR + RT vs. PR + RT) and of SOD-2 and GPx4 in RH30-RR (Fig. 3b, RH30, RR + RT vs. PR + RT). No differences between RMS-PR and -RR were described about the upregulation induced by IR on SOD-2 and GPx4 in RD (Fig. 3b, RD, RR + RT vs. PR + RT) and NRF2 and CAT in RH30 (Fig. 3b, RH30, RR + RT vs. PR + RT). Notably, the basal levels of NRF2 and SOD-2 in RMS-RR, CAT in RD-RR and GPx4 RH30-RR were significantly higher than in the parental counterpart (Fig. 3b, RD and RH30, RR vs. PR). Paralleling the increased antioxidant capacity, 12 h after RT, the expression level of γ-H2AX (a known biomarker of DNA DSBs [24]) resulted lower in irradiated RMS-RR compared to -PR (Fig. 3c, γ-H2AX, RD and RH30, RR + RT vs. PR + RT). This result also suggested that RMS-RR might have a higher ability to repair DSBs. Thus, the activation status of non-homologous end joining (NHEJ) and homologous recombination (HR) DNA repair pathways was investigated. The phosphorylation/activation status of DNA-PKCs and ATM, respectively upstream molecule of NHEJ- and HR pathways, as well as the expression level of their downstream Ku70/Ku80 and RAD51 proteins were assessed by immunoblotting. About NHEJ pathway, the phosphorylation/activation of DNA-PKCs was increased more efficiently by RT (Fig. 3c, DNA-PKCs^PO4^, RD and RH30, RR + RT vs. PR + RT) and resulted basally higher in RMS-RR (Fig. 3c, DNA-PKCs^PO4^, RD and RH30, RR vs. PR). No significance were described on IR-induced accumulation of Ku70 in RH30 (Fig. 3c, Ku70, RH30, RR + RT vs. PR + RT) and Ku80 in RD and RH30 (Fig. 3c, Ku80, RD and RH30, RR + RT vs. PR + RT), with Ku80 that resulted basally higher in RMS-RR cells (Fig. 3c, Ku80, RD and RH30, RR vs. PR). Concerning HR pathway, the phosphorylation/activation of ATM was increased more efficiently by IR (Fig. 3c, ATM^PO4^, RD and RH30, RR + RT vs. PR + RT), whilst the RAD51 accumulation induced by IR in RMS-PR cells was not observed in HR-RMS cells (Fig. 3c, RAD51, RD and RH30, PR + RT vs. RR + RT) even though RAD51 resulted basally higher in RMS-RR than -PR (Fig. 3c, RAD51, RD and RH30, RR vs. PR).

### RMS-RR cells more efficiently than RMS-PR escape from IR-induced cell cycle arrest

**Fig. 4:**
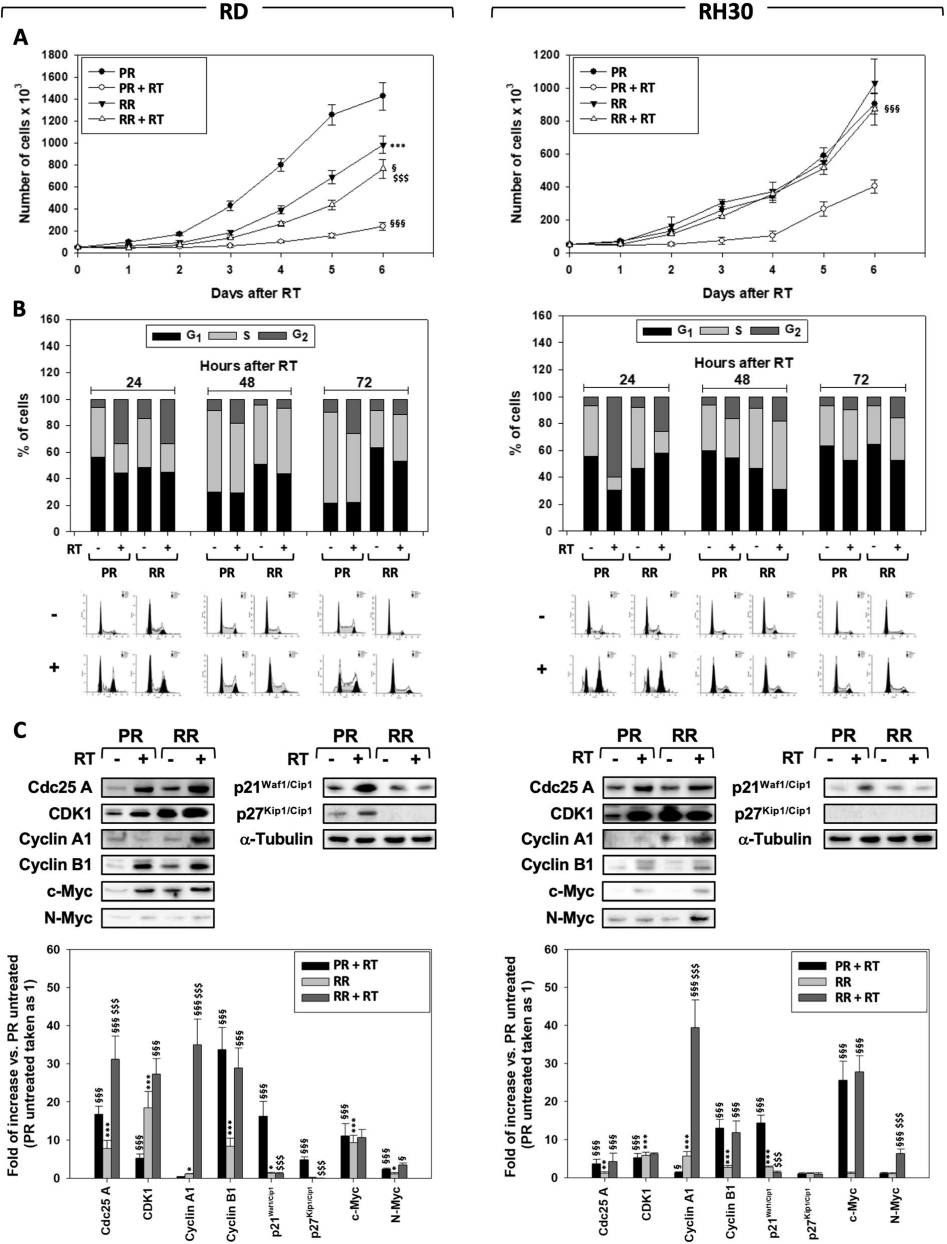
Irradiation differently changes cell cycle distribution in RMS-PR and -RR cells. **a** Effect of irradiation with 6 Gy on cell number of RMS-PR and RMS-RR. **b** FACS analysis performed on RMS-PR and RMS-RR cells after 24, 48 and 72 h from irradiation with 6 Gy. Representative of three different experiments. Results are representative of three different experiments performed in triplicate. **c** Cell lysates from RMS-PR and RMS-RR cells untreated (−) or treated (+) with 6 Gy of irradiation collected 12 h after RT, were analyzed by immunoblotting with specific antibodies for indicated proteins; α-Tubulin expression shows the loading of samples. Western blot showed are representative of three different experiments. Statistical analyses: *p < 0.05, **p < 0.01, ***p < 0.001 RMS-RR NO RT vs. RMS-PR NO RT, ^§^p < 0.05, ^§§^p < 0.01, ^§§§^p < 0.001 RMS-PR RT vs. RMS-PR NO RT and RMS-RR RT vs. RMS-RR NO RT, ^$^p < 0.05, ^$$^p < 0.01, ^$$$^p < 0.001 RMS-RR RT vs. RMS-PR RT.

Unrepaired DSBs induce permanent cell growth arrest or death [3]. Cell growth curve performed on RMS receiving 6 Gy of IR showed that RMS-RR cells recovered IR-induced growth arrest earlier than RMS-PR (Fig. 4a RD and RH30, RR + RT vs. PR + RT). Analysis of the cell cycle by flow cytometry, performed 24, 48 and 72 h after irradiation, showed that IR increased the percentage of cells in the G_2_/M phase more efficiently in RH30-PR compared to RH30-RR (Fig. 4b, RH30, RR + RT vs. PR + RT) and similarly in RD-PR and RD-RR cells (Fig. 4b, RD, RR + RT vs. PR + RT). However, RD-RR cells more quickly than RD-PR escape from G_2_/M growth arrest (Fig. 4b, RD, RR + RT vs. PR + RT, 48 h and 72 h), whilst no difference was observed in RH30-PR and -RR (Fig. 4b, RH30, RR + RT vs. PR + RT, 48 h and 72 h). The expression levels of Cdc25-A, CDK1, Cyclin A1, Cyclin B1, c-Myc and N-Myc positive-, as well as of p21^Waf1/Cip1^ and p27^Kip1/Cip1^, negative-regulator of the G_2_ to M cell cycle transition were assessed. IR upregulated Cdc25-A and Cyclin B1 expression both in RMS-PR and -RR and Cyclin A1 in RMS-RR (Fig. 4c, RD and RH30, − vs. +). CDK1, a natural partner of both Cyclin A1 and B1 proteins, was increased by IR in RMS-PR but not in -RR (Fig. 4c, RD and RH30, RR + RT vs. PR + RT) in which, however, was basally higher compared to RMS-PR (Fig. 4c, RD and RH30, RR vs. PR). RMS-RR significantly counteracted IR-induced p21^Waf1/Cip1^ and p27^Kip1/Cip1^ overexpression (Fig. 4c, RD and RH30, RR + RT vs. PR + RT). IR up-regulated c-Myc expression both in RMS-PR and -RR (Fig. 4c, RD and RH30, RR + RT vs. PR + RT) and resulted basally higher in RD-RR compared to RD-RR (Fig. 4c, RD, RR vs. PR). IR induced the expression of N-Myc in RH30-RR but not -PR (Fig. 4c, RH30, RR + IR vs. PR + IR) and slightly in RD-RR vs. -PR cells (Fig. 4c, RD, RR + RT vs. PR + RT). No differences between RMS-PR and -RR were described about the ability of RT to induce cell death (Additional data 3).

### Cytokine levels and related network in RMS-PR and RR cells

**Fig. 5:**
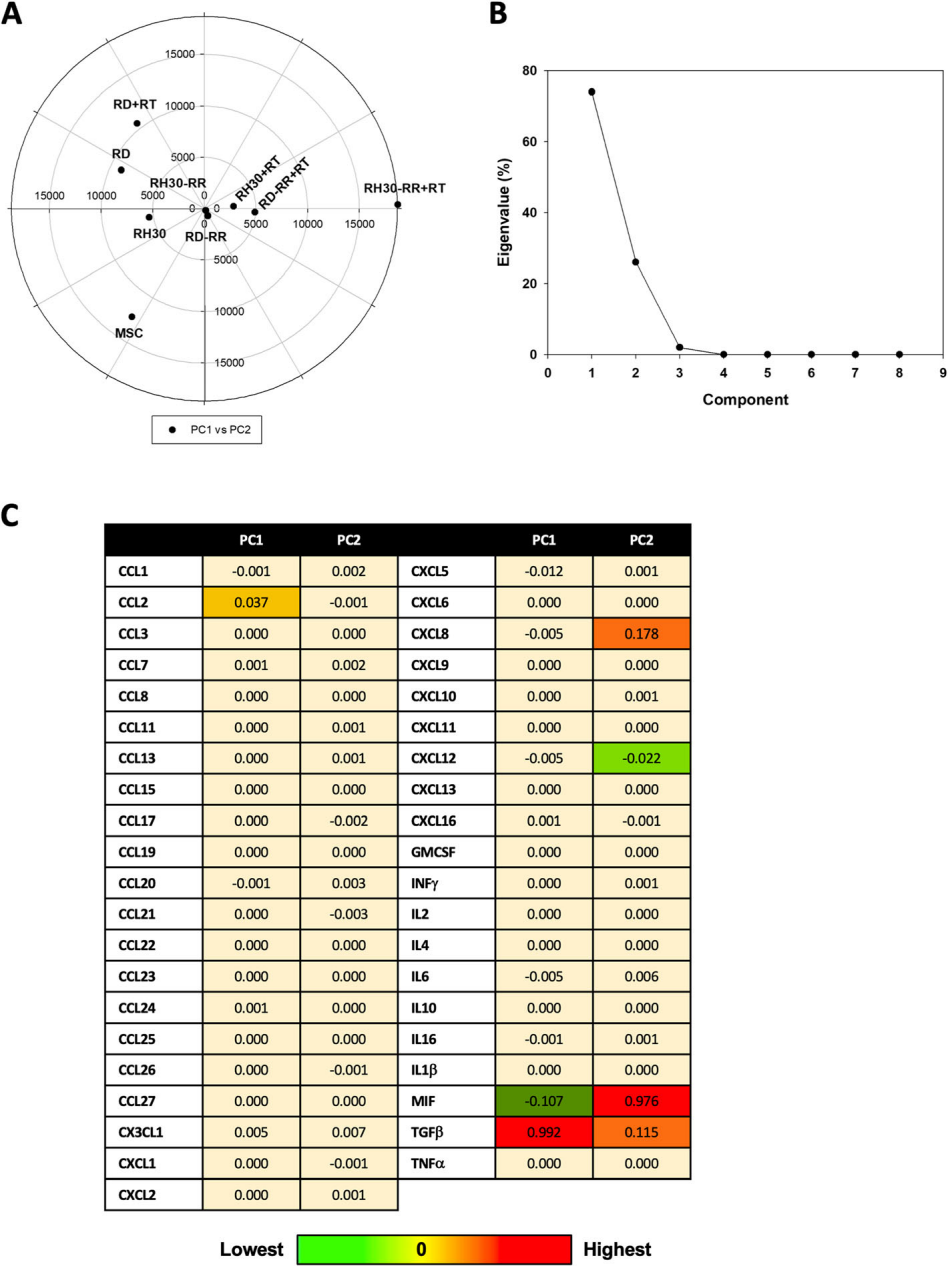
Principal component analysis **a**) PCA analysis: scatter plot of different experimental conditions (MCS, RD, RD + RT, RH30, RH30 + RT, RD-RR, RD-RR + RT, RH30-RR, RH30-RR + RT) based on cytokines concentration. **b**) PCA analysis: scree plot of Principal Components: as it is evident PC1 and PC2 represent about 95% of total variance. **c**) PCA analysis: values of Principal Component 1 and 2 (PC1 and PC2) for the different cytokines. Color ranges from light yellow (lowest value) to red (highest value)

**Fig. 6:**
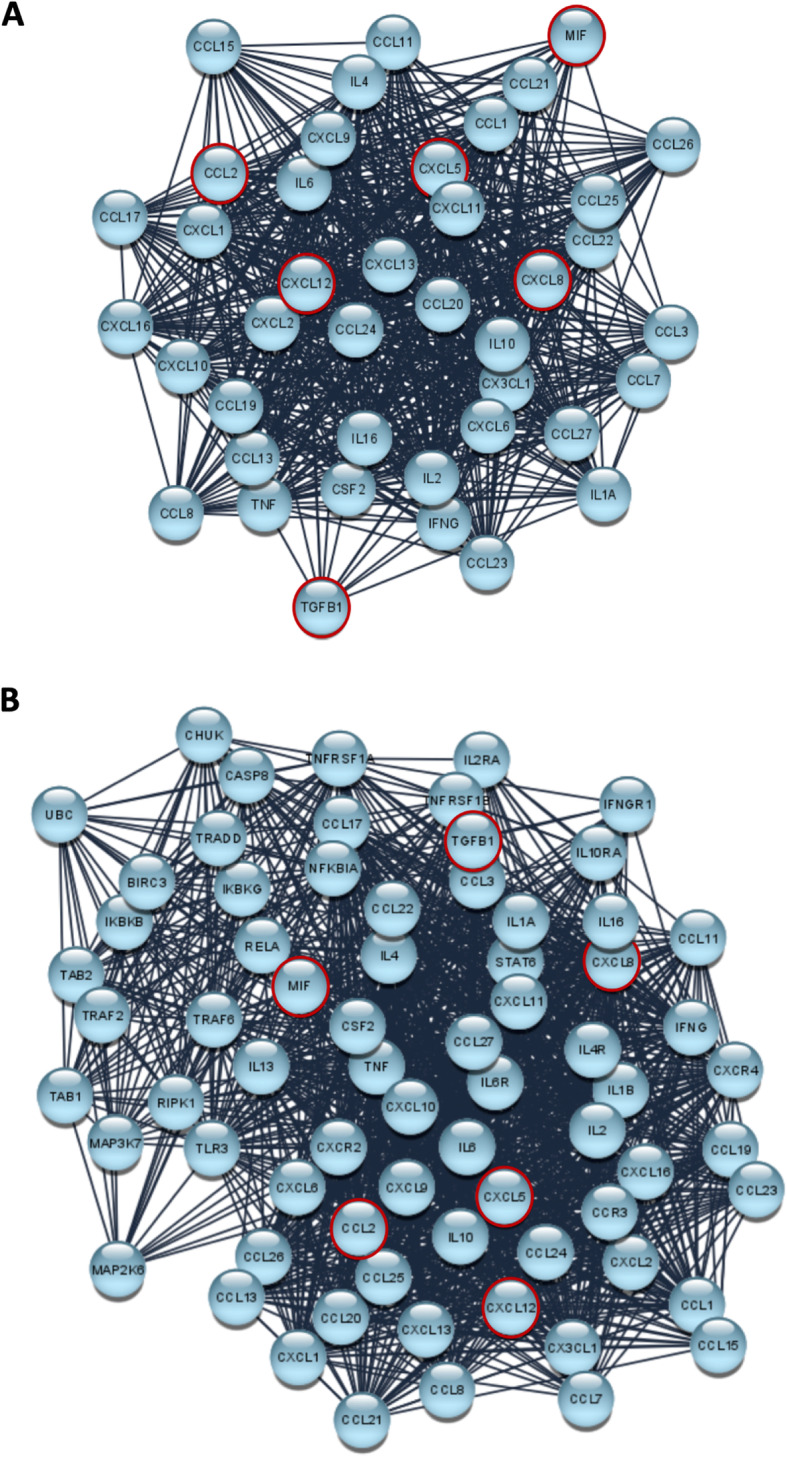
STRING analysis **a**) STRING analysis: CN: network representing the interaction among the examined cytokines (Cytokines Network, CN), as reported in STRING tool. The data are filtered for *Homo sapiens* and with a medium confidence score (0.400). The nodes and links are represented with the default layout and using the edge-weighted spring embedded layout (weighted for edge betweenness). The most relevant nodes as defined by PCA analysis are edged in red. **b**) STRING analysis: previous network after 4 cycles of enrichment (Enriched Cytokines Network, ECN). The nodes and links are represented with the default layout and using the edge-weighted spring embedded layout (weighted for edge betweenness). The most relevant nodes as defined by PCA analysis are edged in red

The expression levels of 41 cytokines involved in cancer development and progression was assessed on cell culture supernatants from mesenchymal (MSC) cells, used as normal counterpart, and RMS-PR and RMS-RR, irradiated or not. Qualitative and quantitative evaluation of basal levels of specific cytokines released by the RMS-PR and -RR cells showed several differences compared to MSC (Additional data 4) and similarly between RMS-PR and -RR after RT (Additional data 5). However, since chemokines are a set of molecules characterized by an integrated network of biological functions, giving rise to the so called “chemokines system”, we performed their evaluation by adopting an unsupervised multivariate statistical tool, the Principal Components Analysis (PCA) [25]. The various experimental conditions (RMS cancer and MCS non-cancer cells), based on cytokine concentrations, were divided in different zones by a typical score plot (Fig. 5a). In particular, MSC are positioned in the left and lower part of the graph; RD-RR, RD-RR + RT, RH30 + RT, and RH30-RR cells constitute a cluster of data relatively similar and characterized by a low variability of cytokine expressing values, whilst RD, RD + RT and RH30, are dispersed in the left quadrants of the graph and, finally, RH30-RR + RT cells are plotted alone in a right quadrant of the graph, this indicating a quite peculiar inflammatory-related pattern. Figure 5b showed the distribution of Principal Components (PC), clearly indicating that PC1 and PC2 represent about 95% of the total variance. Figure 5c shows values of PC1 and PC2 for each specific cytokine, by which TGF-β, MIF, CCL2, CXCL5, CXCL8 and CXCL12 were suggested as key regulators in determining the behavior of examined cancer cells line and particularly in modulating the response to RT of RH30-RR cells. Since the cytokine network has a key role in driving several cell responses by acting as a complex system that also interact with others biochemical entities, we used the STRING analysis tool, a database that includes known and predicted protein-protein interactions, by filtering the data for *Homo sapiens* species, in order to identify and predict new molecules possibly involved in cytokine-related pathways. The specific network, representing the interaction among the 41 examined cytokines – the cytokine network (CN) – was generated (Fig. 6a) as well as after 4 cycles of enrichment by Enriched Cytokines Network (ECN) (Fig. 6b). Protein-protein interactions (PPI) may be either direct (physical) or indirect (functional) associations, and are derived from different sources: genomic context, high-throughput experiments, conserved co-expression, and previous knowledge. Finally, the BiNGO enrichment of ECN was performed and the network representing all the pathways involved was generated and represented in Additional data 6.

## Discussion

The probability of local tumor control after RT remains miserably poor for some tumor types [26], including RMS [1, 2, 14] and understanding the molecular mechanisms driving radioresistance is essential to identify personalized RT-based strategies. At now, the use of cells with different genetic backgrounds and different origins produced data with a low reproducibility in a clinical setting. Thus, increasing evidences indicate that studies should be performed by comparing intrinsic and acquired radioresistance by using cells with the same isogenic background, cells named as clinically relevant radioresistant (RR) [6, 9]. Herein, we presented novel radioresistant cell lines from both ARMS and ERMS subtypes. The inherent differences and responses to RT have been characterized through molecular and cell biology approaches, whilst an immune-related molecular profiling has been performed to understand how these RR cells can escape from RT-induced ICD.

The literature presents RR cell lines generated by using different irradiation schemes [6, 9, 27–32]. Herein, we took inspiration from the hypo-fractionated program used for sarcoma patients, irradiating cells with 6 Gy 6 times [18] that, considering the low α/β ratio of RMS [20], permit us to reach radiobiologically equivalent dose of 66 Gy, equal to that clinically delivered with conventional schedule [2]. It should be noted that fractions were not delivered daily but when cells restarted to growth, as used in other studies [9, 27] that consider tumor cells in 2D more sensitive to treatments [15]. The progressive reduction in the time-intervals between fractions suggested that RMS cells were acquiring radioresistance, as finally confirmed by clonogenic assays, performed 10 passages after the last fraction of 6 Gy. That cell still proliferated resulted more radioresistant after 10 passages confirmed their RR nature [9, 27–32].

It has been already shown that radiation *di* per se and acquired radio-resistance promote a more aggressive and pro-metastatic phenotype [33], as also herein in vitro confirmed in RMS-RR by using different experimental approaches. Multiple biological mechanisms determine radiation-induced metastasis even though increasing evidences indicate the ability of RT to enrich CSCs population as the main mechanisms [33, 34]. Since CSCs cells are more radioresistant, RT, by killing non-CSCs, could promote a relative increase on CSCs number. However, increasing reports provide evidence supporting the idea that non-CSCs exhibit a remarkable degree of plasticity that allows them to re-acquire CSCs traits, in the context of RT. [34] RMS-RR more efficiently formed tumorspheres, previously shown to be enriched in CSCs [20], this suggesting that tumor plasticity could be the main mechanism involved. The evidence that RMS-RR express a more CSC-like phenotype was also indicated by the reduced proliferation rate respect the PR counterpart, as already described in other RR cell lines and addressed to their increased CSCs-like phenotype [6, 9, 27–32]. Moreover, the involvement of both tumor plasticity and CSCs in RT-induced aggressiveness is also suggested by clinical experience on RMS tumors: RT is an efficient treatment in reducing tumor mass, but it is often associated with tumor recurrence due the ability of some cells to resist or become radioresistant. Thus, since RT may lead to a therapeutic failure, it is urgently needed to identify a biology-based tool able to predict response to treatment of cancer patients and identify the most efficient radiosensitizing strategies.

The biology-based stratification of cancer patients in responders and non-responders to RT is based on the radiobiological concept of the “6Rs”, which are repair, redistribution, repopulation, reoxygenation, intrinsic radioresistance and reactivation of antitumor immune response [35]. Thus, we decide to explore each of them in order to identify the molecular mechanisms potentially responsible for intrinsic and acquired radioresistance-related phenomena.

As known, RT induces DNA damage and cell death directly or through the accumulation of intracellular ROS [3]. The activation of DNA damage repair (DDR) [3] pathways and/or ROS detoxifying mechanisms [36] can determine tumor cell survival after RT exposure, including in RMS [21, 22]. Indeed, irradiated RMS-RR cells showed lower levels of γ-H2AX, a biomarker of damaged DNA [24], in comparison mocked PR counterparts, this indicating that rescue mechanisms are involved. About DDR, non-homologous end joining (NHEJ) and homologous recombination (HR), the two major mechanisms of DNA repairing, resulted activated by RT more efficiently in RMS-RR than in PR cells, so suggesting that the DDR activity in could be a surviving strategy in RMS-RR cells. Interestingly, we found that DDR was also basally more activated in RMS-RR and the reasons for this “new setting” of the DDR could be manifold. We speculate that it could be consequent to the high stem-like cell tracts showed by RR cell lines as also indicated by consolidated evidences of the prompt activation of DNA damage sensor and repair machinery by CSCs able to survive to stressful events [7, 23]. Moreover, accordingly to our recent findings showing the efficient anti-oxidant system possessed by RMS cells [21, 22], herein, we also found that RMS-RR are able to detoxify from ROS as RMS-PR, but the expression of key molecular drivers, such as NRF2, CAT, SOD-2 and GPx4, is more quickly and efficiently activated in RH30-RR than in PR cells. Interestingly, also the basal expression levels of these enzymes resulted higher in RR than PR, as frequently showed in CSCs and so in line with the increased stemness already suggested. Thus, the detoxifying abilities of RMS suggest that the use of anti-oxidants by RMS patients during RT could be deleterious. The use of pro- or anti-oxidant molecules during RT is a controversial item since whether some evidence suggests that anti-oxidants may improve tumor response and patient survival, whilst others, opposite effect [37]. Clarifying the role of anti- or pro-oxidants adjuvants during RT in RMS is another topic of considerable importance which will be the subject of future investigations. Interestingly, despite RMS-RR showing greater anti-oxidant and repair ability for genomic damage, the data showed no difference in RT-induced apoptosis between RMS-PR and -RR. Accumulating evidences suggest that induction of apoptosis alone is insufficient to account for the therapeutic effect of RT. Thus, the inhibition of the proliferative capacity of malignant cells following irradiation, especially with solid tumors, can occur via alternative cell death modalities, including permanent cell cycle arrests [38]. In our case, the fact that the irradiated RMS-RRs maintain a high proliferative rate unlike the RMS-PR, which only begin to proliferate again after a few days from the RT, suggests just such a mechanism. Future experiments will also be performed to verify the possible intervention of further mechanisms, including senescence and autophagy.

Redistribution refers to the ability of RT to restrain tumor cells in the G_2_/M high radio-responsiveness phase of the cell cycle in order to permit a higher efficiency of subsequent fractions [35]. Thus, radioresistant cancer cells are expected to restrain G_2_/M induced arrest and escape from this constriction. This event occurred in both RMS-RR cell lines, but was differently induced in RD and RH30, with RD-RR that came out from G_2_/M arrest faster than RD-PR and RH30-RR counteracting G_2_/M. To this concern, RD-RR and RH30-RR cells seem to use a partially common molecular approach based on the modulation of different cell cycle regulators. Differently to RMS-PR, both irradiated RD-RR and RH30-RR expressed Cyclin A and restrained RT-induced p21^Waf1/Cip1^ upregulation. Cyclin A regulates the transition from the late S phase to the late G_2_/M phase when it is replaced by cyclin B [39], whilst p21^Waf1/Cip1^, in addition to the G1 block, can also contribute to a delay in G2 by inhibiting Cyclin A and B1 dependent kinase activity and then replicative DNA synthesis [40]. Thus, RMS-RR cells could boost G_2_/M transition by promoting and inhibiting signals, which are able to regulate this phase positively or negatively, respectively. This hypothesis is also supported by the fact that RMS-RR basally expressed higher levels of CDK1, a natural partner of both Cyclin A1 and B1, and upregulated after RT the expression of Cyclin B1, a strategic protein in the G_2_ to M transition. Notably, a role seems to be suggested also for c-Myc and N-Myc oncogenes, known to be key regulators of the cell cycle [41] and respectively known to sustain the transformed phenotype of ERMS [42, 43] and ARMS [44, 45]. Herein, RD-RR basally expressed higher levels of c-Myc whilst RH30-RR up-regulated N-Myc expression after RT. On the other hand, RT significantly induced the expression of c-Myc in RH30 and N-Myc in RD, independently from their radio-sensitivity. Thus, in line with already collected evidences showing that the overexpression of Myc family members attenuates DNA damage-induced G2/M arrest [46], we suppose that also the modulation of c-Myc and N-Myc could participate in the maintenance of intrinsic and acquired radio-resistance. Notably, the fact that c-Myc seem to also has a role in ARMS and N-Myc in ERMS is in line with other evidences [47, 48] suggest as the family member of MYC family could interplay to sustain oncogenesis.

Tumor repopulation by surviving cells after fractionated RT and intrinsic radioresistance as well as the different sensitivity of cancer cells to radiation have been shown to be related to CSC population [35]. Indeed, CSCs represent one of the most important elements that determine local tumor control and CSCs are intrinsically more radioresistant than non-CSCs. Accordingly, our results confirm that both the repopulation ability and the intrinsic radioresistance are improved in RMS-RR cell lines by their increased stemness features. However, in vivo experiments will be performed to better characterize these phenomena, as well as re-oxygenation.

Notably, cancer stem cells have been shown to be chemoresistant [49] and, more generally, radioresistance and chemoresistance are closely related [50, 51], thus suggesting that RMS-RR could be more chemoresistant than the RMS-PR counterpart. CHT, as RT, kills cancer cells preferentially by apoptosis [52], whose molecular regulators commonly sustain both chemo- and radio-resistance [52]. Herein, collected evidences do not show differences on RT-induced apoptosis between RMS-RR and -PR, indicating that, in RMS, apoptosis is not the master key regulator of RT-induced death and suggesting that RMS-RR could be not more chemoresistant. Future experiments will be carried out in this sense, in vitro and in vivo, also considering that the chemotherapy for RMS is based on the use of multiple drugs.

In the context of reactivation of antitumor immune response linked to the ability of RT to induce immunogenic cell death (ICD) [4], cytokines play a key role in mediating the host-response against cancer cells by guiding leukocytes trafficking into the tumor microenvironment [5]. Indeed, chemokine expression has an important role in the immune system response, and their dysregulation is implicated in tumor repopulation through sustained radioresistance mechanisms [5]. Notwithstanding, also tumor cells are able to secerns cytokines and a balance between “good” and “bad” chemokines have been demonstrated to be essential in cancer biology and response to conventional therapies, especially RT. Our RMS-RR cell lines represent an in vitro system to deep insight the immunomodulatory response induced by RT in tumor cells in order to better understand the molecular and biological events that are critical in the radioresistance mechanisms. Indeed, many studies have demonstrated that the secretion of specific cytokines act as regulators of the immune suppression within the tumor microenvironment and to have a negative effect on RT ability to generate an in situ tumor vaccine [5], so we investigated the expression of 41 chemokines, differently involved in the relationship between cancer and immune system, in RMS-PR and -RR cell lines, irradiated or not. The evaluation of these immunomodulatory factors has shown similarities and differences, both quantitative and qualitative, between normal mesenchymal cells and RMS cancer cells as well as between RMS-PR and -RR cell lines. In order to dissect the complicate chemokine interactions and their biological function in tumor cells, mainly in RR phenotype, we integrated our molecular and expression data with a bioinformatic approach. Specifically, we used the principal components analysis (PCA), an unsupervised multivariate statistical analysis, able to simplify the complexity in high-dimensional data while retaining trends and patterns [19], to assess the overall state of the immune-related network in RMS cells by enhancing the role of those interrelated cytokines having a specific expression pattern after RT exposure. Our study indicated that TGF-β, MIF, CCL2, CXCL5, CXCL8 and CXCL12 are key players of both intrinsic and acquired radioresistance. The obtained results indicate that chemokines are differently expressed in RMS malignant and MSC non-malignant cells, but also among the RMS-PR and RR cell lines and considering the RT exposure, this confirming the high heterogeneity among ARMS and ERMS subtypes. In particular, PCA indicates that RH30-RR + RT cells have specific biological characteristics with aberrant levels of inflammatory factors, which are involved in radioresistent mechanisms, this having a potential clinical significance. Notably, TGF-β, which promotes tumor growth in different neoplasia [53], including RMS [54], acts as a negative master regulator of RT-induced direct cell death and ICD, by respectively inducing DNA damage recognition and repair as well as IR-induced in situ tumor vaccination [55]. Similarly, MIF, which is a pleiotropic cytokine frequently overexpressed in many cancer types, is able to promote tumor growth and progression by protecting cancer cells from ICD [56]. MIF is released by cancer cells during RT and, even though its role remains largely understood, the pro-oncogenic profile of MIF suggests its role in mitigating the beneficial effects of RT. [54] Same pro-oncogenic role has been shown for CXCL8 [57]. Concerning CCL2, this chemokine is produced by cancer cells and is correlated with monocytes infiltration into the tumor site, this resulting in enhanced metastatic potential [58]. CXCL5 acts as a protumor molecule in different cancer types and it is associated with neutrophil trafficking, cancer angiogenesis, progression and resistance to therapies [59]. Finally, CXCL12 [60] has shown to directly promote radioresistance of several cancer types by different mechanisms, including sustaining stemness and inhibiting immunoresponse.

Although further investigations are needed, these six cytokines resulting from our analysis, seems to well represent the general characteristics of RMS, enhanced in the RR phenotype. TGF-β and MIF, CCL2, CXCL5, CXCL8 and CXCL12 seem might represent the fulcrum of an autocrine/paracrine system able to: i) promote repair of damaged DNA and increase cell proliferation; ii) induce angiogenesis and enhance metastatic potential; iii) protect tumor cells from RT-induced ICD; iv) promoting stemness. Thus, despite the need for further studies, it is suggestive to hypothesize that these selected cytokines may represent potential targets for new radio-sensitized strategies as well as being used as predictive markers of response to RT.

## Conclusions

In conclusion, our study describes the various steps for the establishment of RMS-RR cell lines by analyzing biological, molecular and immune-related features. Moreover, our bioinformatic approach also demonstrates that PCA is a useful tool for describing complex and interrelated data, such as the expression of a panel of cytokines, which thus may represent novel diagnostic markers and/or potential targets for setting tailored and more efficient adjuvant radio-therapeutic strategies in the treatment of patients with RMS.

## Supplementary information

**Additional file 1: Additional data 1** Topological parameters assessed in this study.

**Additional file 2: Additional data 2** Pro-angiogenic abilities of RMS-PR and RMS-RR cell lines. A) HUVECs were seeded in Matrigel in media generated by 96 h incubation with RMS-PR or -RR cells. Cells were photographed 16 h after plating. B) Cell lysates from HUVEC, untreated or treated with media generated by 96 h incubation with RMS-PR or -RR cells, were analyzed by immunoblotting with specific antibodies for indicated proteins; α-Tubulin expression shows the loading of samples. Western blot showed are representative of three different experiments.

**Additional file 3: Additional data 3** Radiation-induced apoptosis is not significantly affected by RMS-PR or -RR phenotype. RMS-PR and -RR cell lines were treated or not with a dose of 6 Gy of radiation and the percentage of viable, apoptotic and necrotic cells assessed by Annexin V assay 12 h later. Images shows data from three independent experiments performed in triplicate (Upper Panel) Lower panel shows results from a representative experiment.

**Additional file 4: Additional data 4** Characterization and identification cytokines release from RMS-PR and RMS-RR cell lines compared to normal mesenchymal cells. Panel of 41 cytokine was assessed in cell culture supernatants from RMS-PR and RMS-RR, 24 h after plating and compared to normal mesenchymal cells (MSC) taken as 1. Panels show cytokines detected and/or modulated. Statistical analyses: *p < 0.05, **p < 0.01, ***p < 0.001 RMS-PR vs. MSC, ^§^p < 0.05, ^§§^p < 0.01, ^§§§^p < 0.001 RMS-RR vs. PR, ^$^p < 0.05, ^$$^p < 0.01, ^$$$^p < 0.001 RMS-RR vs. RMS-PR.

**Additional file 5: Additional data 5** Characterization and identification cytokines release from RMS-PR and RMS-RR cell lines after irradiation. Panel of 41 cytokine was assessed in cell culture supernatants from RMS-PR and RMS-RR, 24 h after irradiation (6 Gy) and compared to non-irradiated counterpart taken as 1. Panels show cytokines detected and/or modulated. Statistical analyses: *p < 0.05, **p < 0.01, ***p < 0.001 RMS-PR RT vs. RMS-PR NO RT, ^§^p < 0.05, ^§§^p < 0.01, ^§§§^p < 0.001 RMS-RR RT vs. RMS-RR NO, ^$^p < 0.05, ^$$^p < 0.01, ^$$$^p < 0.001 RMS-RR RT vs. RMS-PR RT.

**Additional file 6: Additional data 6** STRING analysis with BINGO enrichment of ECN. For network topology and node description, see Supporting Material 2. The node size depends on the node degree (number of links per node) and the color depends on the *p*-value (Additional data 7).

**Additional file 7: Additional data 7**

## Data Availability

Analytic methods and study materials will be made available on publication of this research article. The data will be available from the corresponding author on reasonable request.

## Abbreviations

RT: Radiotherapy
RMS: Rhabdomyosarcoma
RR: Clinically relevant radioresitant
PR: Parental
ARMS: Alveolar rhabdomyosarcoma
ERMS: Embryonal rhabdomyosarcoma
CHT: Chemotherapy
IR: Ionizing Radiations
DSBs: DNA Double strand breaks
ICD: Immunogenic cell death
CSCs: Cancer stem cells
MSC: Mesenchymal stromal cells
ROS: Reactive oxygen species
NHEJ: Non-homologous end joining
HR: Homologous recombination
PCA: Principal component analysi
DDR: DNA damage repair
EQ D2: Equivalent dose
CN: Cytokines network
ECN: Enriched cytokines network
