# Supplementary material for: Clinically relevant radioresistant rhabdomyosarcoma cell lines: functional, molecular and immune-related characterization
Source: J Biomed Sci. 2020 Aug 27;27:90. doi: 10.1186/s12929-020-00683-6 (PMC7453562; doi:10.1186/s12929-020-00683-6)
Supplement: Supplementary file 1 — Additional file 1: Additional data 1 Topological parameters assessed in this study. [file 12929_2020_683_MOESM1_ESM.pdf]

Additional data 1

Main topological parameters assessed in network analyses.

Parameter	Definition
Connected Components	It is the number of networks in which any two vertices are connected to each other by links, and which is connected to no additional vertices in the network.
Number of nodes	It is the total number of molecules involved.
Number of edges	It is the total number of interactions found.
Clustering coefficient	It is calculated as $C_I = 2n_I/k_I(k_I–1)$, where n_I is the number of links connecting the k_I neighbors of node I to each other. It is a measure of how the nodes tend to form clusters.
Network diameter	It is the longest of all the calculated shortest paths in a network.
Shortest paths	The length of the shortest path between two nodes *n* and *m* is *L*(*n*,*m*). The **s**hortest path length distribution gives the number of node pairs (*n*,*m*) with *L*(*n*,*m*) = *k* for *k = 1,2,…*
Characteristic path length	It is the expected distance between two connected nodes.
Averaged number of neighbors	It is the mean number of connections of each node.
Node degree	It is the number of interactions of each node.
Node degree distribution	It represents the probability that a selected node has *k* links.
γ	Exponent of node degree equation.
R^2	Coefficient of determination of node degree vs. number of nodes, on logarithmized data.

In addition, the following measures were computed as follows:

The **betweenness centrality**[1] $C_b(n)$ of a node *n* is computed as follows:

$C_b(n) = \sum_{s \neq n \neq t} (\sigma_{st}(n) / \sigma_{st})$,

where *s* and *t* are nodes in the network different from *n*, σ_{st} denotes the number of shortest paths from *s* to *t*, and $\sigma_{st}(n)$ is the number of shortest paths from *s* to *t* that *n* lies on.

Betweenness centrality is computed only for networks that do not contain multiple edges. The betweenness value for each node *n* is normalized by dividing by the number of node pairs excluding *n*: $(N-1)(N-2)/2$, where *N* is the total number of nodes in the connected component that *n* belongs to. Thus, the betweenness centrality of each node is a number between 0 and 1.

The **closeness centrality**[2] $C_c(n)$ of a node *n* is defined as the reciprocal of the average shortest path length and is computed as follows:

$C_c(n) = 1 / avg(L(n,m))$,

where $L(n,m)$ is the length of the shortest path between two nodes n and m. The closeness centrality of each node is a number between 0 and 1.

NetworkAnalyzer computes the closeness centrality of all nodes and plots it against the number of neighbors. The closeness centrality of isolated nodes is equal to 0.

Closeness centrality is a measure of how fast information spreads from a given node to other reachable nodes in the network[2].

The **topological coefficient**[3] T_n of a node n with k_n neighbors is computed as follows: $T_n = avg\ (J(n,m))/k_n$.

Where, $J(n,m)$ is defined for all nodes m which share at least one neighbor with n. The value $J(n,m)$ is the number of neighbors shared between the nodes n and m, plus one if there is a direct link between n and m.

The topological coefficient is a relative measure for the extent to which a node shares neighbors with other nodes[4].

Commentato [FM1]: Metterei una lista delle referenze alla fine della tabella senza inserirle nella bibliografia del main text. Solitamente con i Suppl Mat si fa così

References in Suppl Table 1

1. Brandes, U. A faster algorithm for betweenness centrality. J Math Sociol 2001;25:163-177.
2. Newman MEJ. A measure of betweenness centrality based on random walks. arXiv 2003 cond-mat/0309045.
3. Maslov S, Sneppen K. Specificity and stability in topology of protein networks. Science 2002;296:910-913.
4. Stelzl U, Worm U, Lalowski M, et al. A human protein-protein interaction network: a resource for annotating the proteome. Cell 2005;122:957-968.
