# Supplementary figures and images for: Clinically relevant radioresistant rhabdomyosarcoma cell lines: functional, molecular and immune-related characterization
Source: J Biomed Sci. 2020 Aug 27;27:90. doi: 10.1186/s12929-020-00683-6 (PMC7453562; doi:10.1186/s12929-020-00683-6)

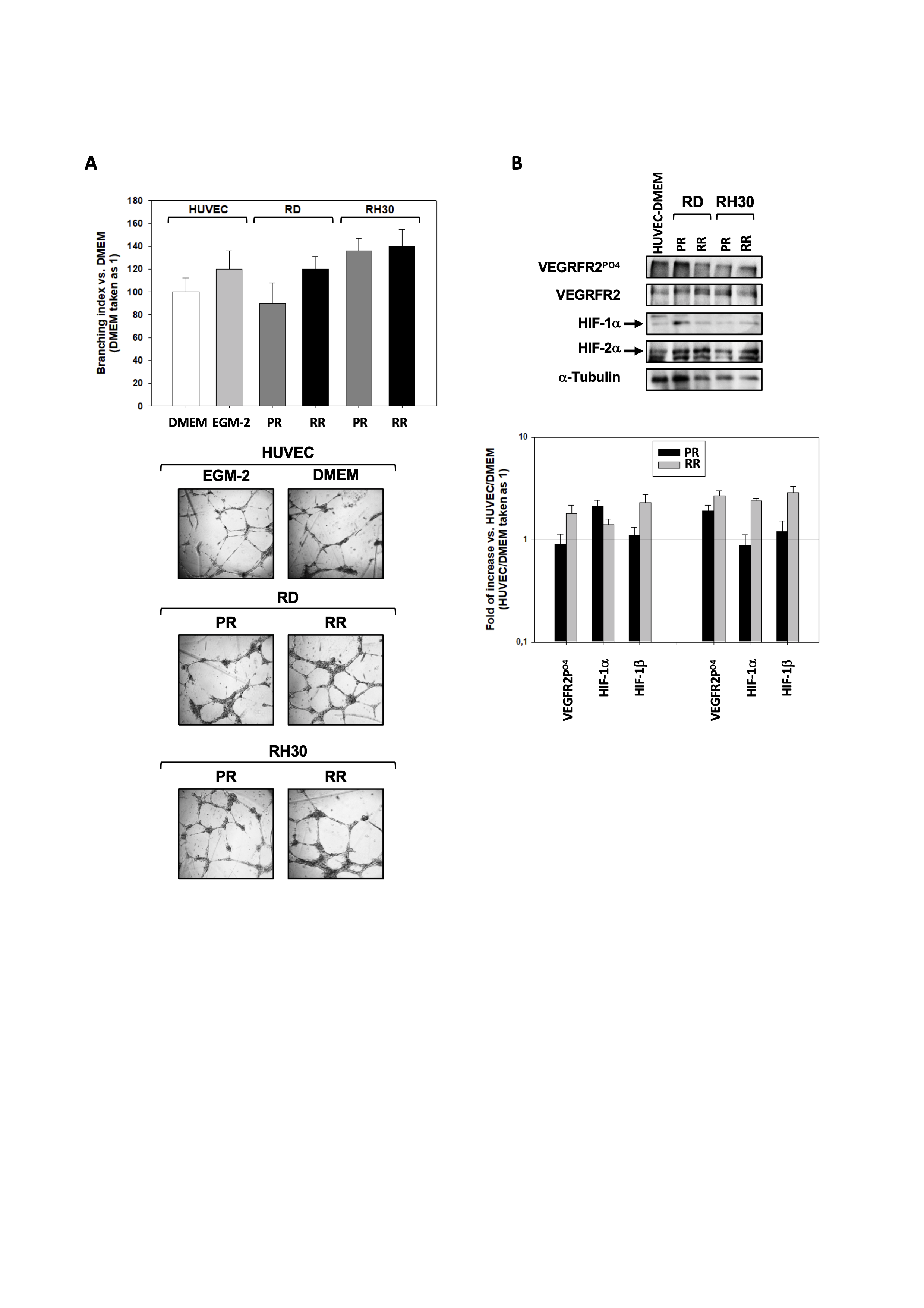

Supplement: Supplementary file 2 — Additional file 2: Additional data 2 Pro-angiogenic abilities of RMS-PR and RMS-RR cell lines. A) HUVECs were seeded in Matrigel in media generated by 96 h incubation with RMS-PR or -RR cells. Cells were photographed 16 h after plating. B) Cell lysates from HUVEC, untreated or treated with media generated by 96 h incubation with RMS-PR or -RR cells, were analyzed by immunoblotting with specific antibodies for indicated proteins; α-Tubulin expression shows the loading of samples. Western blot showed are representative of three different experiments. [file 12929_2020_683_MOESM2_ESM.tiff]

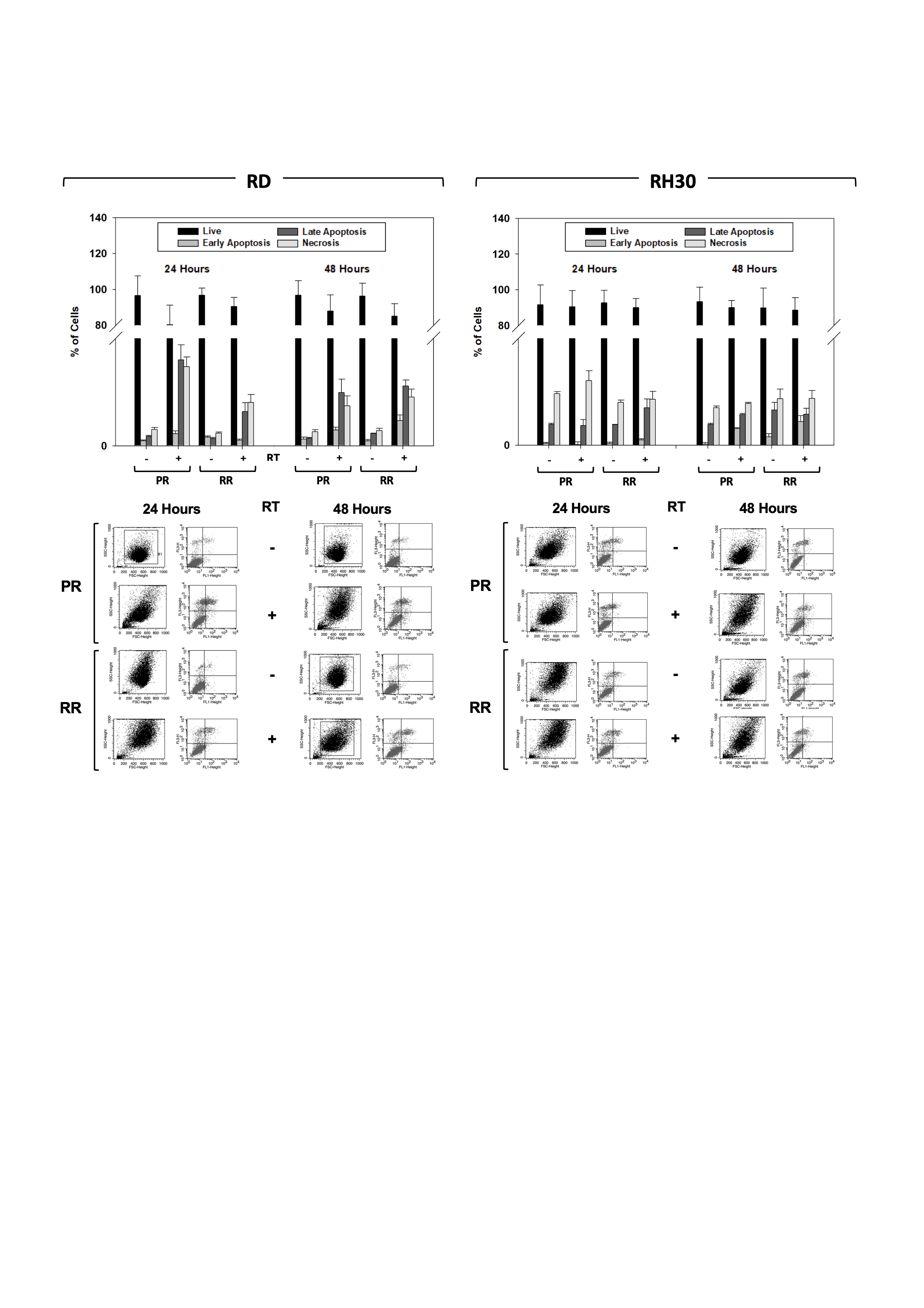

Supplement: Supplementary file 3 — Additional file 3: Additional data 3 Radiation-induced apoptosis is not significantly affected by RMS-PR or -RR phenotype. RMS-PR and -RR cell lines were treated or not with a dose of 6 Gy of radiation and the percentage of viable, apoptotic and necrotic cells assessed by Annexin V assay 12 h later. Images shows data from three independent experiments performed in triplicate (Upper Panel) Lower panel shows results from a representative experiment. [file 12929_2020_683_MOESM3_ESM.tiff]

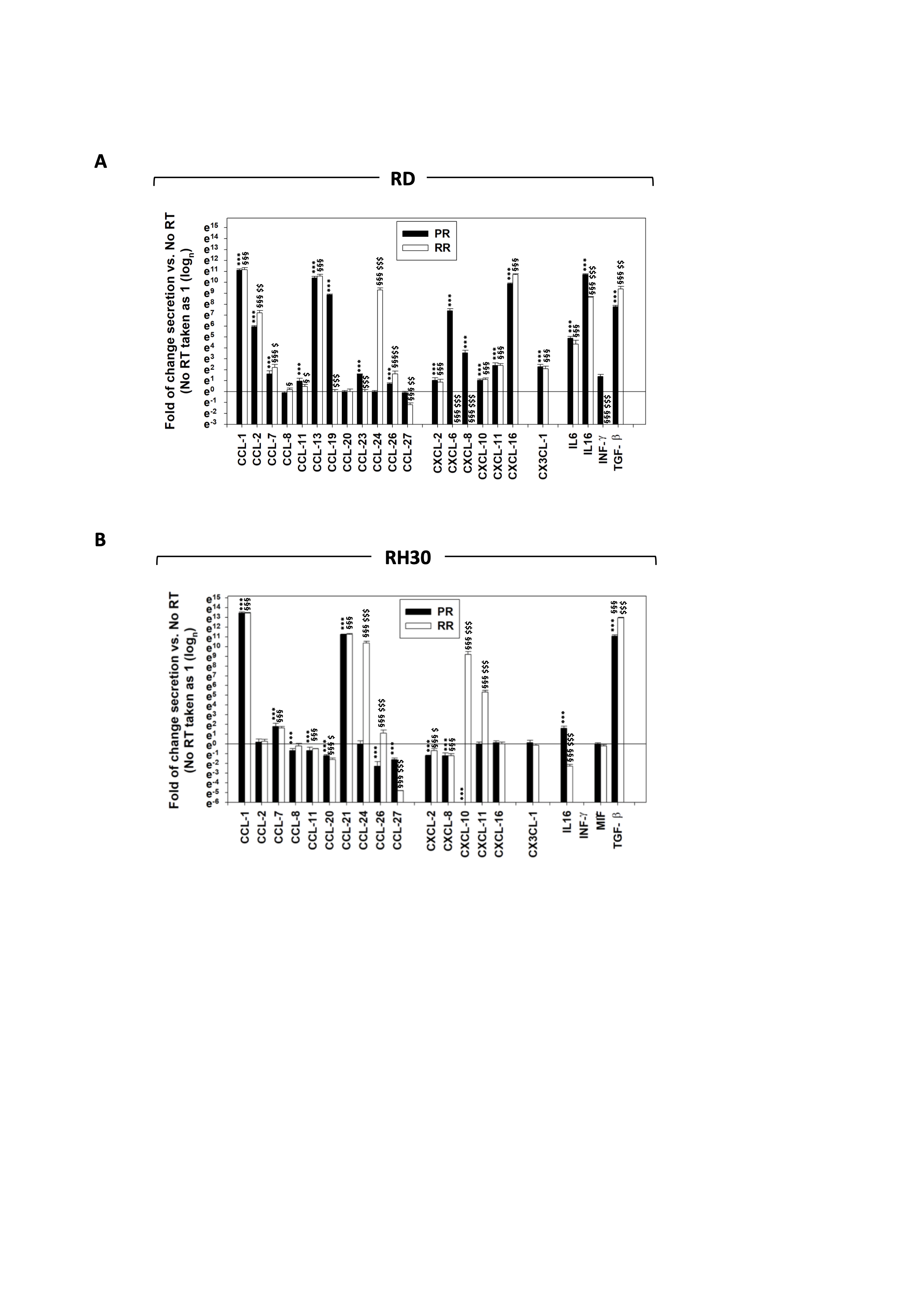

Supplement: Supplementary file 4 — Additional file 4: Additional data 4 Characterization and identification cytokines release from RMS-PR and RMS-RR cell lines compared to normal mesenchymal cells. Panel of 41 cytokine was assessed in cell culture supernatants from RMS-PR and RMS-RR, 24 h after plating and compared to normal mesenchymal cells (MSC) taken as 1. Panels show cytokines detected and/or modulated. Statistical analyses: *p < 0.05, **p < 0.01, ***p < 0.001 RMS-PR vs. MSC, §p < 0.05, §§p < 0.01, §§§p < 0.001 RMS-RR vs. PR, $p < 0.05, $$p < 0.01, $$$p < 0.001 RMS-RR vs. RMS-PR. [file 12929_2020_683_MOESM4_ESM.tiff]

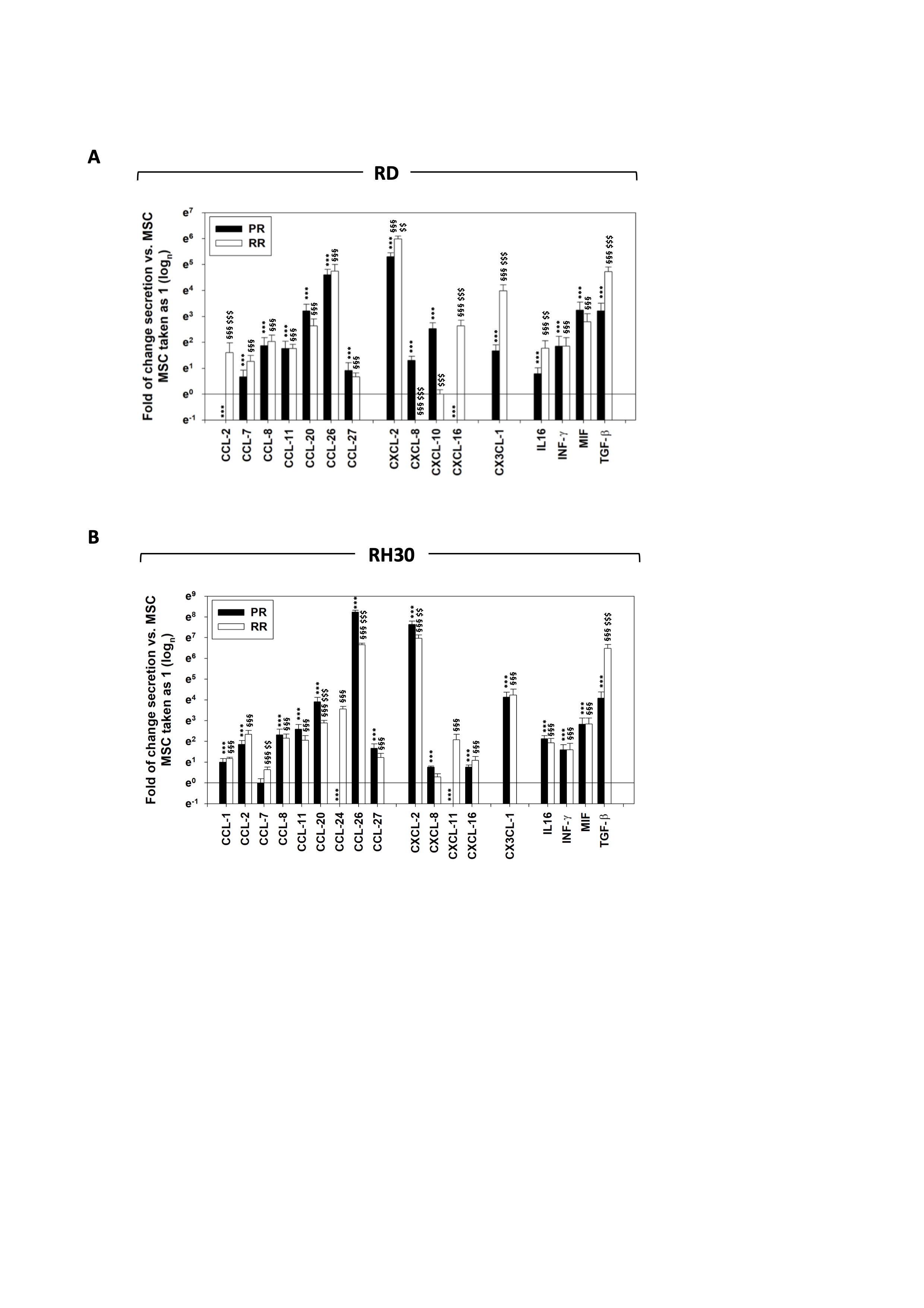

Supplement: Supplementary file 5 — Additional file 5: Additional data 5 Characterization and identification cytokines release from RMS-PR and RMS-RR cell lines after irradiation. Panel of 41 cytokine was assessed in cell culture supernatants from RMS-PR and RMS-RR, 24 h after irradiation (6 Gy) and compared to non-irradiated counterpart taken as 1. Panels show cytokines detected and/or modulated. Statistical analyses: *p < 0.05, **p < 0.01, ***p < 0.001 RMS-PR RT vs. RMS-PR NO RT, §p < 0.05, §§p < 0.01, §§§p < 0.001 RMS-RR RT vs. RMS-RR NO, $p < 0.05, $$p < 0.01, $$$p < 0.001 RMS-RR RT vs. RMS-PR RT. [file 12929_2020_683_MOESM5_ESM.tiff]

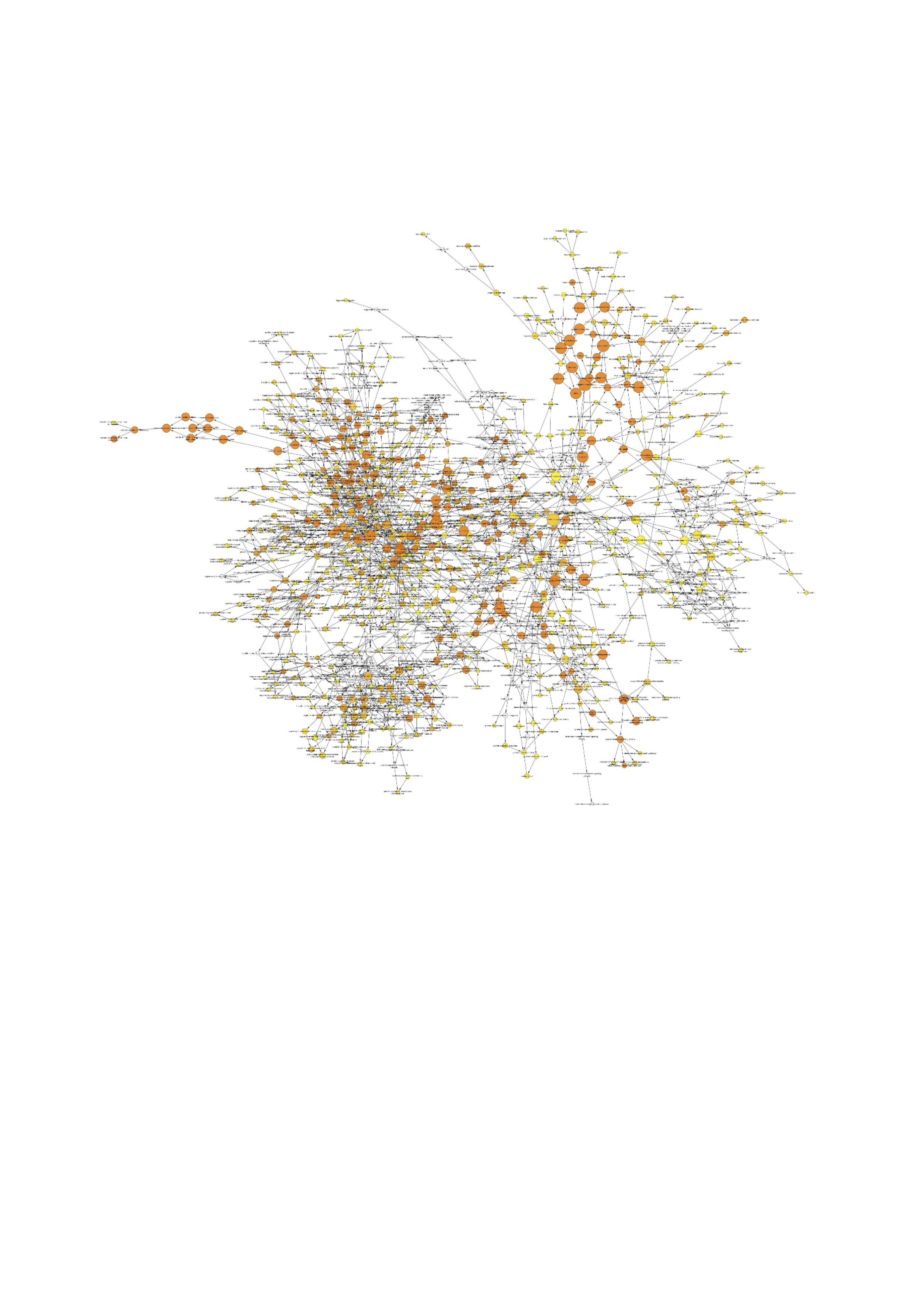

Supplement: Supplementary file 6 — Additional file 6: Additional data 6 STRING analysis with BINGO enrichment of ECN. For network topology and node description, see Supporting Material 2. The node size depends on the node degree (number of links per node) and the color depends on the p-value (Additional data 7). [file 12929_2020_683_MOESM6_ESM.tiff]
